# Supplementary material for: APOE4 and INPP5D converge on membrane mechanics to regulate endocytosis in human astrocytes
Source: bioRxiv. 2026 May 22:2026.05.22.726347. Preprint. [Version 1] doi: 10.64898/2026.05.22.726347 (PMC13228211; doi:10.64898/2026.05.22.726347)
Supplement: 1 [file NIHPP2026.05.22.726347v1-supplement-1.pdf]

bioRxiv preprint doi: https://doi.org/10.64898/2026.05.22.726347; this version posted May 25, 2026. The copyright holder for this preprint (which was not certified by peer review) is the author/funder, who has granted bioRxiv a license to display the preprint in perpetuity. It is made available under a CC-BY 4.0 International license.

SUPPLEMENTAL FIGURE 1

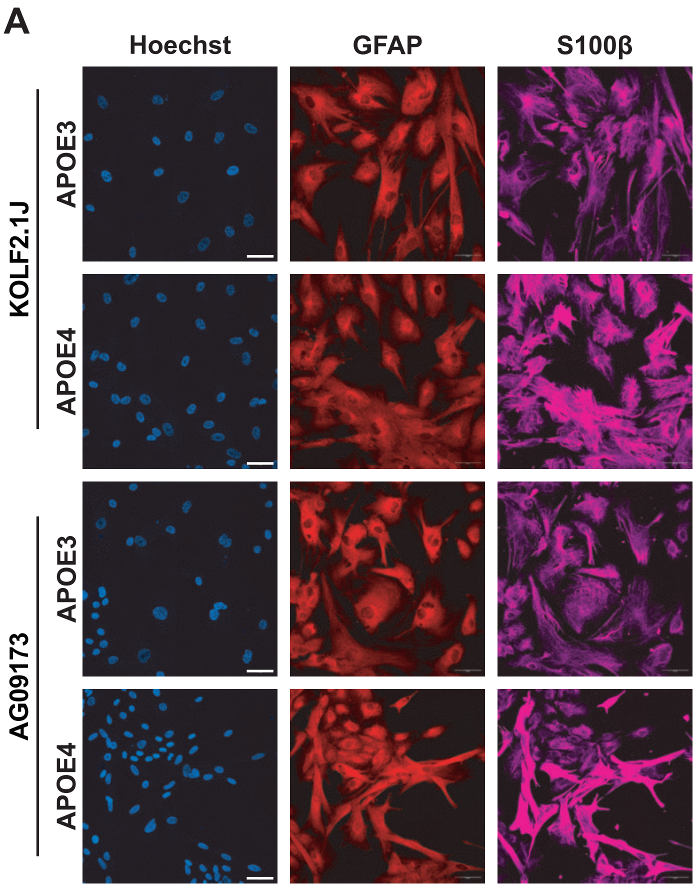

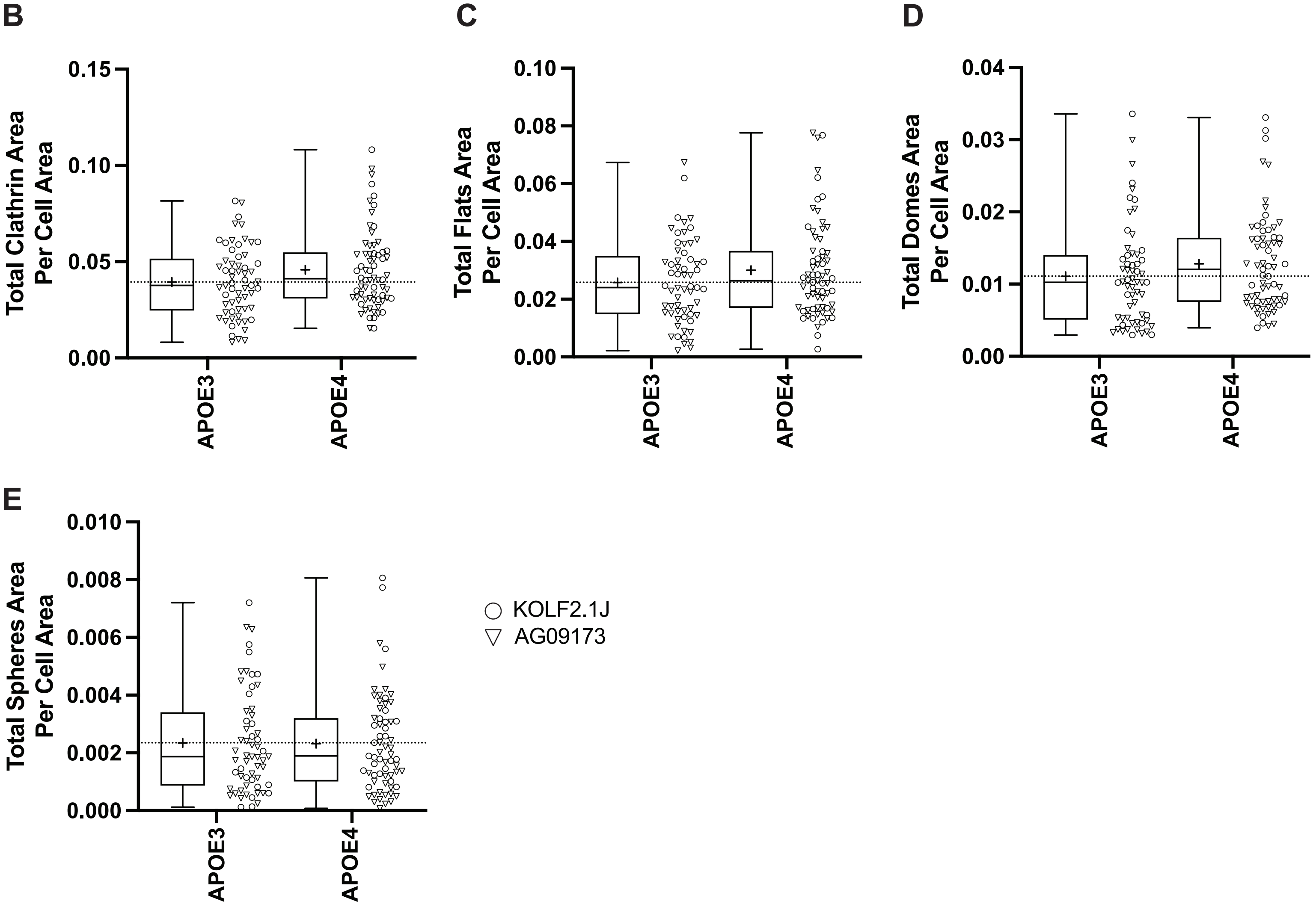

bioRxiv preprint doi: https://doi.org/10.64898/2026.05.22.726347; this version posted May 25, 2026. The copyright holder for this preprint (which was not certified by peer review) is the author/funder, who has granted bioRxiv a license to display the preprint in perpetuity. It is made available under a CC-BY 4.0 International license.

Supplemental Figure 1: iPSC line quality control and further PREM characterization.

A) GFAP and S100β immunocytochemistry for KOLF2.1J and AG09173 iPSC-derived astrocytes (*APOE3* and *APOE4* homozygous lines). Scale bar is 50 μm. Quantification of **B)** total clathrin area, **C)** total flat area, **D)** total dome area, and **E)** total sphere area as a function of cell membrane area in membranes imaged with PREM. N=61-68 cells per genotype, across two iPSC lines (KOLF2.1 O, AG09173 ∇). Box and whisker, minimum to maximum with line at median, cross (+) denotes mean alongside individual cell data. Unpaired t-test used for statistical analysis.

bioRxiv preprint doi: https://doi.org/10.64898/2026.05.22.726347; this version posted May 25, 2026. The copyright holder for this preprint (which was not certified by peer review) is the author/funder, who has granted bioRxiv a license to display the preprint in perpetuity. It is made available under a CC-BY 4.0 International license.

SUPPLEMENTAL FIGURE 2

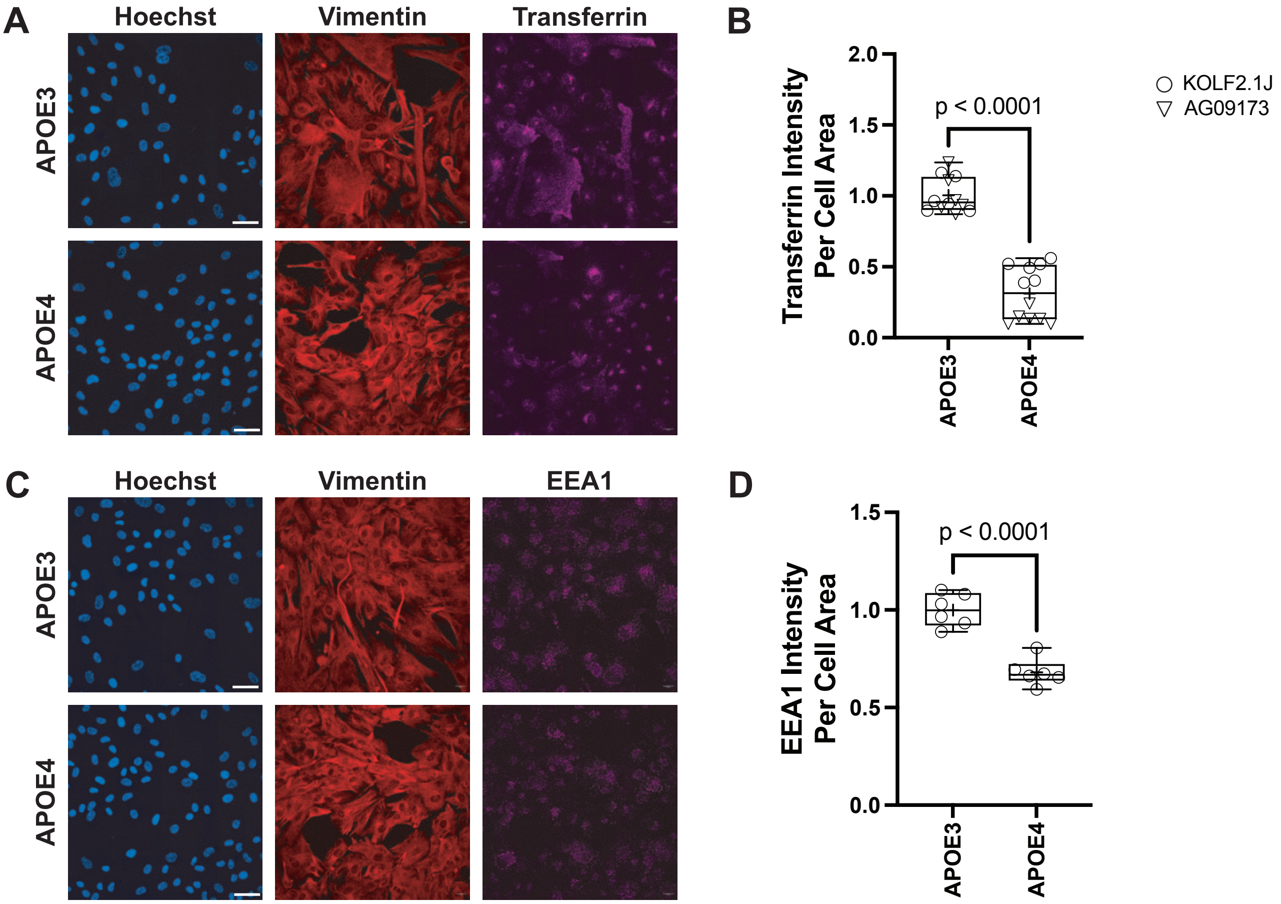

bioRxiv preprint doi: https://doi.org/10.64898/2026.05.22.726347; this version posted May 25, 2026. The copyright holder for this preprint (which was not certified by peer review) is the author/funder, who has granted bioRxiv a license to display the preprint in perpetuity. It is made available under a CC-BY 4.0 International license.

Supplemental Figure 2: Immunofluorescence images of transferrin uptake and EEA1 endosomes in iPSC-derived astrocytes

Representative images for **A)** cellular uptake of fluorescent transferrin with **B)** quantification of fluorescent intensity per cell, N=12 wells per genotype, across two iPSC isogenic lines (KOLF2.1 O, AG09173 ▽). Representative images for **C)** EEA1 positive early endosomes with **D)** quantification (N=6 wells per genotype for KOLF2.1J). Box and whisker, minimum to maximum with line at median, cross (+) indicates mean. Unpaired t-test used for statistical analysis. All scale bars are 50 μm.

bioRxiv preprint doi: https://doi.org/10.64898/2026.05.22.726347; this version posted May 25, 2026. The copyright holder for this preprint (which was not certified by peer review) is the author/funder, who has granted bioRxiv a license to display the preprint in perpetuity. It is made available under a CC-BY 4.0 International license.

SUPPLEMENTAL FIGURE 3

A

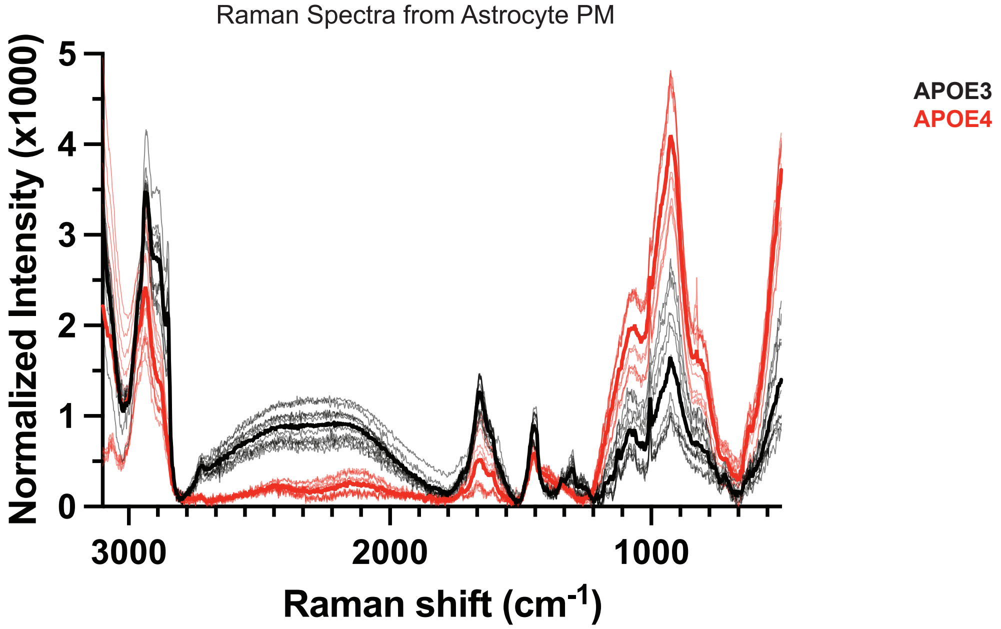

bioRxiv preprint doi: https://doi.org/10.64898/2026.05.22.726347; this version posted May 25, 2026. The copyright holder for this preprint (which was not certified by peer review) is the author/funder, who has granted bioRxiv a license to display the preprint in perpetuity. It is made available under a CC-BY 4.0 International license.

Supplemental Figure 3: Complete Raman spectra from *APOE3* and *APOE4* astrocyte plasma membrane regions.

A) Full Raman spectrum from 500 cm^{-1} to 3100 cm^{-1}, from N=10 membrane regions from *APOE3* astrocytes (grey, solid black = *APOE3* average) and from N=10 membrane regions from *APOE4* astrocytes (pink, solid red = *APOE4* average)

bioRxiv preprint doi: https://doi.org/10.64898/2026.05.22.726347; this version posted May 25, 2026. The copyright holder for this preprint (which was not certified by peer review) is the author/funder, who has granted bioRxiv a license to display the preprint in perpetuity. It is made available under a CC-BY 4.0 International license.

SUPPLEMENTAL FIGURE 4

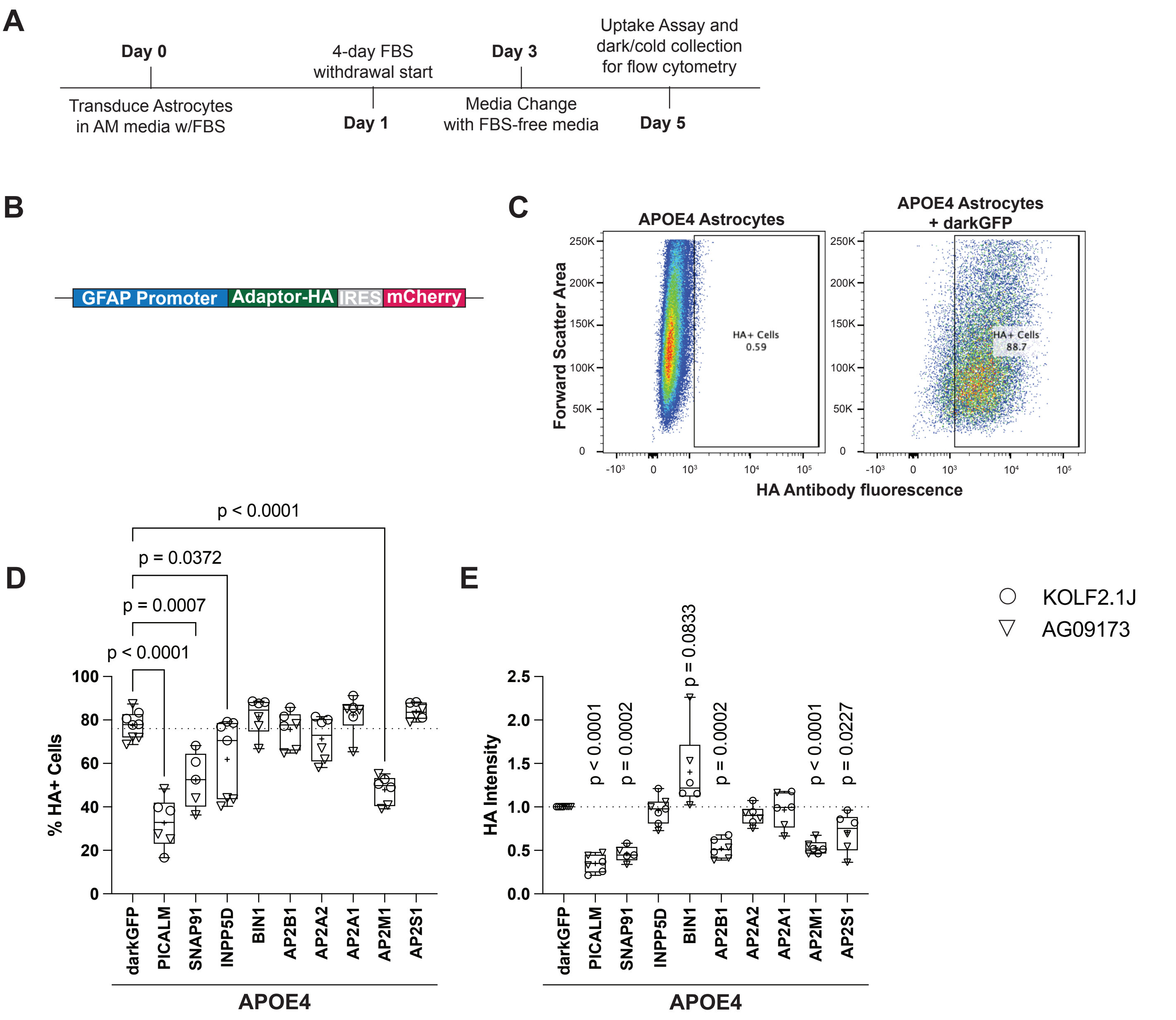

bioRxiv preprint doi: https://doi.org/10.64898/2026.05.22.726347; this version posted May 25, 2026. The copyright holder for this preprint (which was not certified by peer review) is the author/funder, who has granted bioRxiv a license to display the preprint in perpetuity. It is made available under a CC-BY 4.0 International license.

Supplemental Figure 4: Exogenously expressed protein expression levels

A) Experimental workflow for *APOE4* astrocyte lentiviral transduction and culture in serum free media before endocytosis quantification via flow cytometry. **B)** Diagram of lentiviral construct used to transduce and express each candidate gene of interest (GOI) tagged with HA in *APOE4* astrocytes. **C)** Gating example to identify positively transduced *APOE4* astrocytes based on HA positivity. Box and whisker plot (minimum to maximum values) for quantification of multiple flow cytometry runs for **D)** percentage of transduced cells (quantified by number of singlet cells that are HA positive), N=5-7 flow cytometry runs per endocytosis protein, across two iPSC lines (KOLF2.1J, AG09173), one-way ANOVA with multiple comparisons used for statistical analysis. **E)** HA tag intensity from positively transduced astrocytes, N=5-7 independent flow cytometry runs per endocytosis protein, across two iPSC lines (KOLF2.1 O, AG09173 ▽) flow cytometry means normalized to *APOE4* astrocytes transduced with control *darkGFP* values from same run, Wilcoxon signed-rank test.

bioRxiv preprint doi: https://doi.org/10.64898/2026.05.22.726347; this version posted May 25, 2026. The copyright holder for this preprint (which was not certified by peer review) is the author/funder, who has granted bioRxiv a license to display the preprint in perpetuity. It is made available under a CC-BY 4.0 International license.

SUPPLEMENTAL FIGURE 5

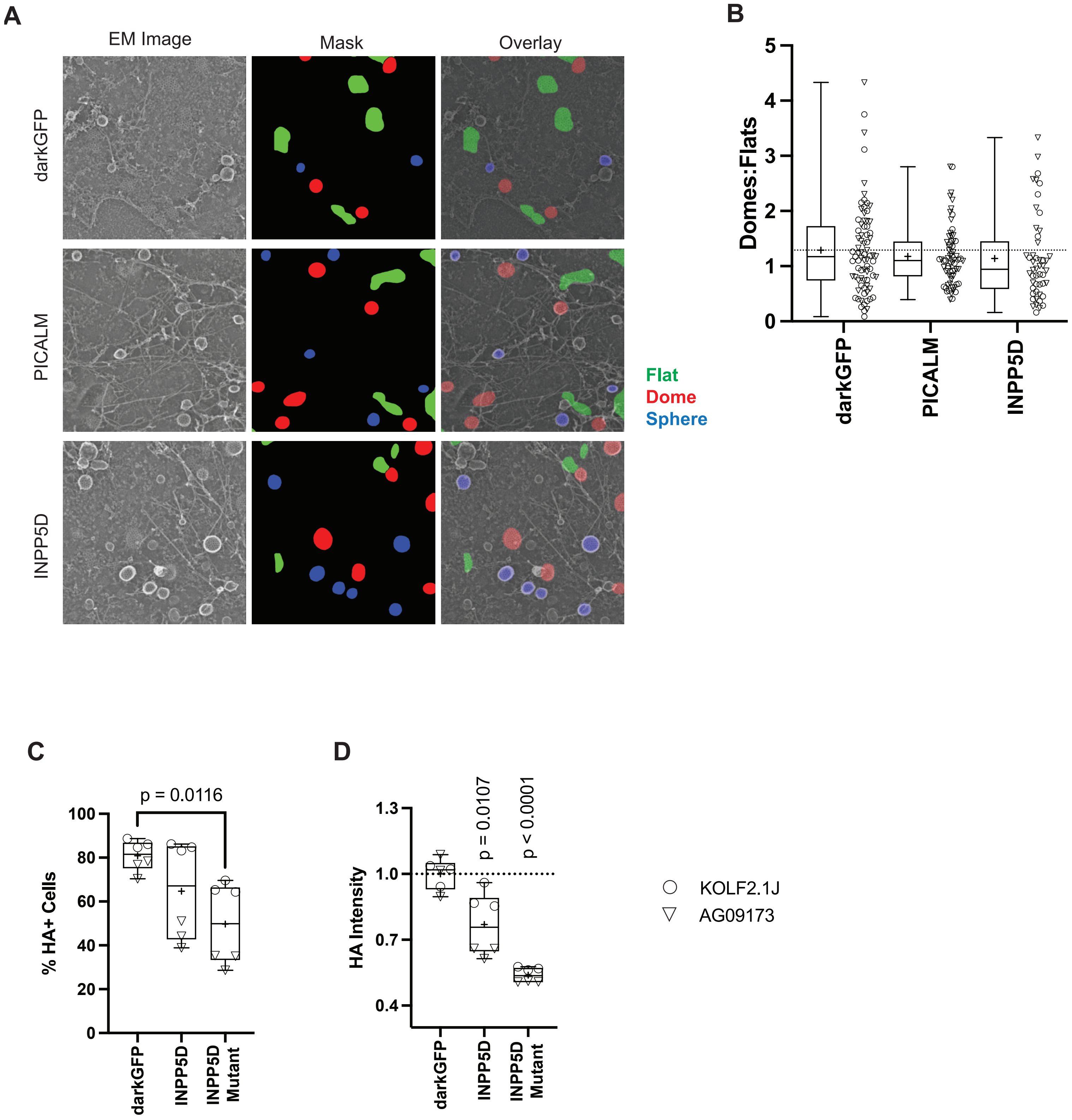

bioRxiv preprint doi: https://doi.org/10.64898/2026.05.22.726347; this version posted May 25, 2026. The copyright holder for this preprint (which was not certified by peer review) is the author/funder, who has granted bioRxiv a license to display the preprint in perpetuity. It is made available under a CC-BY 4.0 International license.

Supplemental Figure 5: Adaptor PREM and mutant expression levels

A) Example images of PREM from *darkGFP*, *PICALM*, or *INPP5DI*, with flat, dome, and spherical clathrin structure masks used for quantifications. **B)** PREM quantification of number of Domes:Flats ratio in *darkGFP*, *PICALM*, or *INPP5D* expressing *APOE4* astrocytes. N=44-81 cells per condition, across two iPSC isogenic lines (KOLF2.1 O, AG09173 ∇). Box and whisker, minimum to maximum with line at median, cross (+) indicates mean. One-way ANOVA with multiple comparisons used for statistical analysis. **C)** Box and whisker plot (minimum to maximum values with line at median) for quantification of multiple flow cytometry runs for percentage of transduced cells (quantified by number of singlet cells that are HA positive), N=6 flow cytometry runs per condition, across two iPSC lines (KOLF2.1J, AG09173), one-way ANOVA with multiple comparisons used for statistical analysis. **D)** HA tag immunofluorescence intensity from positively transduced astrocytes, N=6 flow cytometry runs per condition, across two iPSC lines (KOLF2.1 O, AG09173 ∇) flow cytometry means normalized to *APOE4* astrocytes transduced with control *darkGFP* values from same run, Wilcoxon signed-rank test.
